# Tavistock Adult Depression Study (TADS): a randomised controlled trial of psychoanalytic psychotherapy for treatment-resistant/treatment-refractory forms of depression

**DOI:** 10.1186/1471-244X-12-60

**Published:** 2012-07-12

**Authors:** David Taylor, Jo-anne Carlyle, Susan McPherson, Felicitas Rost, Rachel Thomas, Peter Fonagy

**Affiliations:** 1 Department of Clinical, Educational and Health Psychology, University College London, London, UK; 2 Adult Department, Tavistock & Portman NHS Foundation Trust, London, UK; 3 School of Health and Human Sciences, University of Essex, Colchester, UK; 4 Psychology, Psychotherapy, Consultancy and Training in the Community (PSYCTC), Hamilton House, Mabledon Place, London, UK

## Abstract

**Background:**

Long-term forms of depression represent a significant mental health problem for which there is a lack of effective evidence-based treatment. This study aims to produce findings about the effectiveness of psychoanalytic psychotherapy in patients with treatment-resistant/treatment-refractory depression and to deepen the understanding of this complex form of depression.

**Methods/Design:**

INDEX GROUP: Patients with treatment resistant/treatment refractory depression. DEFINITION & INCLUSION CRITERIA: Current major depressive disorder, 2 years history of depression, a minimum of two failed treatment attempts, ≥14 on the HRSD or ≥21 on the BDI-II, plus complex personality and/or psycho-social difficulties. EXCLUSION CRITERIA: Moderate or severe learning disability, psychotic illness, bipolar disorder, substance dependency or receipt of test intervention in the previous two years. DESIGN: Pragmatic, randomised controlled trial with qualitative and clinical components. TEST INTERVENTION: 18 months of weekly psychoanalytic psychotherapy, manualised and fidelity-assessed using the Psychotherapy Process Q-Sort. CONTROL CONDITION: Treatment as usual, managed by the referring practitioner. RECRUITMENT: GP referrals from primary care. RCT MAIN OUTCOME: HRSD (with ≤14 as remission). SECONDARY OUTCOMES: depression severity (BDI-II), degree of co-morbid disorders Axis-I and Axis-II (SCID-I and SCID-II-PQ), quality of life and functioning (GAF, CORE, Q-les-Q), object relations (PROQ2a), Cost-effectiveness analysis (CSRI and GP medical records). FOLLOW-UP: 2 years. Plus: a). Qualitative study of participants’ and therapists’ problem formulation, experience of treatment and of participation in trial. (b) Narrative data from semi-structured pre/post psychodynamic interviews to produce prototypes of responders and non-responders. (c) Clinical case-studies of sub-types of TRD and of change.

**Discussion:**

TRD needs complex, long-term intervention and extended research follow-up for the proper evaluation of treatment outcome. This pushes at the limits of the design of randomised therapeutic trials. We discuss some of the consequent problems and suggest how they may be mitigated.

**Trial registration:**

Current Controlled Trials ISRCTN40586372

## Background

Worldwide, depressive disorders have consistently been shown to be the largest contributor to the burden of human disease [1,2]. This is connected with the fact that depression tends to pursue chronic or relapsing courses. In primary care samples in the UK, 25% to 40% of patients presenting with an index depression have at least one further episode within the next two years; and within 5 years, 60% will have had at least one further episode [3]. On average, three-quarters of those who have had a depressive illness will suffer four further episodes. While most depressive episodes tend to last about 3 months, in about 12% of patients episodes last for longer than 2 years. These figures suggest that something like 0.7% to 1.0% of the general adult population suffer from long-term disabling depression. Depressed patients may also respond only partially to treatment or may withdraw from it prematurely. Of those showing these sub-optimal therapeutic responses, Stimpson [4] estimated that a minimum of 30% of patients experience recurrent treatment failures. The clinical management problems they can pose led to the suggestion of the existence of a distinct ‘treatment-resistant’ or ‘treatment-refractory’ form of depression. There are no findings to suggest that a single pathogenic mechanism underlies these difficult-to-help conditions, and the 2010 update to the NICE Depression Guideline rejected the use of the treatment-resistant (or treatment-refractory) diagnosis. Here we simply employ it as a shorthand to denote an operationally defined, clinically significant, but heterogeneous group of patients.

### Research evidence

These disabling, incompletely recovering and long-term forms of depression are increasingly recognised to be a significant mental health problem in both primary and secondary care settings [5-10]. However until very recently, there has been a shortage of research to guide the clinical management of patients with these disorders [4,11]. This shortage of evidence is also linked to the way that these depressions are long-term, relapsing and complex [12]. Co-morbidity with other common mental disorders is the rule rather than the exception [13,14]. Difficulties with psychosocial functioning hinder important help-seeking and illness-combating behaviours: moreover the patient’s disorder can have negative effects on service providers and individual caregivers [15,16]. There is accumulating evidence to suggest that to be effective, treatments for these depressions have themselves to be both more complex and longer than required for simpler disorders [17]. These requirements strain the testing capacities of randomised controlled therapeutic trials. To determine properly the effectiveness of a given treatment for a disorder which is both chronic and relapsing, observation needs to be continued over a long follow-up period. This is expensive and difficult to sustain [18]. In spite of these problems, recent research has begun to bear upon the needs of patients with these types of depression, and to examine the complex services and treatments required [9,19-21]. Ideally, future studies should combine pragmatic randomised trial designs with the exploratory possibilities offered by qualitative research methods [22-25].

### Psychoanalytic psychotherapy for depression (PPD)

PPD is a complex intervention based upon psychoanalytic theories of the nature and origins of depression [26,27]. It aims to help patients alter key aspects of personal functioning, often connected with developmentally early experiences of loss, to reduce an underlying depressive diathesis [28]. As Stiles, Shapiro and Firth-Cozens [29] argue, the mode of action of this category of therapy is fundamentally different from that of physical interventions such as drugs, for which randomised controlled trials were designed. For example, the usual dose–response algorithms do not apply. However, outcome research findings do in fact offer support for the effectiveness of psychoanalytically-based treatments for depression. Evidence from RCTs indicates that short forms of psychodynamic therapy may be as effective in reducing depressive symptoms as medication or other short forms of psychological therapy (for example cognitive behaviour therapy [CBT]) [20,30-33]. As well, there are cohort and observational studies which suggest that more durable additional benefits may accrue from longer-term or intensive psychoanalytic treatments [34-37]. These longer treatments are based on the idea that patients are gradually able to internalise a psychological capacity which enables them to relate to pathogenetic personal experiences, memories, feelings, beliefs and relationships in a more reflective, yet also more active way [38]. Findings from developmental, observational, genetic and neuroscientific studies also offer support for some of the main theses of the psychoanalytic account of depression [39,40].

Over the last fifty years there has gradually developed an adaptation of psychoanalytic ideas to a once weekly psychotherapy format for use in public sector conditions. Versions of this adaptation have been used in many UK publicly-funded psychodynamic psychotherapy services. Although widely adopted, their effectiveness has been under-investigated, as is the case for all longer term psychotherapies [41].

## Design & methods

### The hypotheses

As detailed below, the primary hypothesis is that PPD is superior in effectiveness to a comunity treatment as usual control group (TAU) using the Hamilton Depression Rating Scale as its primary outcome measure. The study tests the effectiveness of psychoanalytic therapy with a research sample of patients suffering from TRD, as an exacting test of its hypothesised ability, both to reduce symptoms over the immediate term, and to yield significant reductions in long-term susceptibility to depression. The trial has been designed to test the effectiveness of the PPD model as it might be offered in the context of an ordinary psychotherapy clinic, to patients with long term mental health problems including major depression. The aim therefore was to stay as close as possible to the setting in which those patients with chronic TRD may be referred, and to offer the therapy in a form as little modified as possible, while meeting what is required by trial methodology. A pilot study of two cases was conducted before commencing the study proper, to obtain information about the practicalities of both the therapeutic and the research enterprises.

We employ an intention-to-treat methodology with all eligible, consenting participants. The participants are randomised into two groups: one receives a target of 60 sessions of once-weekly psychoanalytic psychotherapy for depression (PPD) over a period of eighteen months [in practice the actual number is allowed to vary slightly according to factors such as the timing of holidays], while the other represents a ‘treatment as usual’ (TAU) control condition. The PPD group is assessed over the trial intervention and for a two year follow-up. The TAU control group is observed over the same 3.5 year period.

### The study centre

All patients attended the Adult Department of the Tavistock and Portman NHS Foundation Trust in North London, whether for measurement alone or for the test treatment and measurement. The Department (and the Tavistock Clinic of which it is a part) is an important provider of psychoanalytic psychotherapy in the UK’s publicly-funded National Health Service. The Clinic has a distinguished record of providing human-relations and applied psychoanalytic approaches to mental health in the public setting. The research team are staff members of the Tavistock and Portman’s Psychotherapy Evaluation Research Unit (PERU). Research assessments are conducted in a designated office of the Tavistock Clinic.

### The sample

#### *The definition of TRD*

A patient is defined as having TRD when there has been a minimum of 2 years’ history of depression, assessed via SCID-I, plus at least two failed previous attempts at treatment (elicited at interview and verified from medical records). At least one of these must have included treatment with an antidepressant medication (ADM), and the other(s) either with an ADM, or a psychological intervention. Most patients also meet Axis-II criteria for other diagnoses. These are assessed using data from a clinical interview schedule - the Tavistock Dynamic Interview (TDI) - an instrument primarily concerned with representations of self and interpersonal relationships. However, the material is suitable for the Westen-Shedler Q-sort [42], and for the application of DSM-5 criteria [43].

#### *Sample size, power calculation & primary outcome*

Because the analysis of the expected primary outcome is concerned with testing superiority of PPD relative to a comunity treatment control group (TAU), we employ an 80% one-sided confidence interval (CI) to assess potential non-inferiority. The trial is powered to detect a 35% difference in effectiveness between PPD and TAU on the Hamilton Depression Rating Scale HRSD-17 [44] with alpha at 0.05 and beta at 0.9. The primary outcome for treatment efficacy is the rating of depressive symptoms by an objective assessor blind to treatment allocation. Estimating effect-size on the basis of previous trials summarized in meta-analyses using the HDRS-17 in psychosocial treatments, with or without pharmacological therapies (e.g. [45]), we could achieve the target levels of alpha and beta with 80 cases randomised equally into two arms. However, this does not take into account within-therapist correlations. Based on statistical analysis of data from another trial of long term psychodynamic therapy with a similarly heterogeneous population [46] we have conservatively assumed an intra-cluster correlation coefficient for therapists of 0.05. With a minimum of 10 therapists delivering each therapy, each seeing on average 5 subjects, the study will have 80% power to reject inferiority with a non-infidelity margin equal to an effect size of 0.5 using an 80% one-sided confidence interval, on the basis of a 90% follow-up rate to 6 months. Given the pragmatic focus of the trial, the degree of adherence to treatment protocols is of considerable interest. The main analyses will be based on an intention-to-treat and complementary “as treated” bases.

#### *Recruitment & the determination of eligibility*

Participants are recruited from general practitioners (GPs) based in the Primary Care Trusts (PCTs) of central and north London (including some PCTs on the outskirts of these areas). In all, 425 practices have been approached, with 119 agreeing to refer. Each is sent a pack containing information sheets for referrers and participants, eligibility criteria, referral forms, leaflets, and posters for the waiting area. In addition, members of the research team visit practices to discuss the trial, TRD and the test treatment.

Participating practices then refer potential participants whom they consider meet the eligibility criteria (see Table 1).

**Table 1:** TADS Eligibility Criteria

**Patient Eligibility Criteria**	
**Inclusion Criteria**	**Exclusion Criteria**
Major Depressive Disorder or Dysthymia	Recent (previous five years) history of psychosis
Minimum of a two-year history of depression	Recent (previous five year) history of bi-polar disorder
At least two previous failed treatment attempts, one of which was with an anti-depressant	Moderate or severe learning disabilities
Age 18 – 65	Recent history (previous two years) of psychiatric input for, or diagnosis of, substance dependency (alcohol abuse ≥21 units/week; drug abuse ≥ 4/week)
Able to speak conversational English and be seen at the Tavistock Clinic London	Patients currently in psychological therapy
Willing to enter a randomised control trial	Patients who have received psychoanalytic psychotherapy in the previous two years

Two research interviews are then used to verify eligibility criteria and to confirm that these referrals in fact meet DSM-IV criteria for Major Depressive Disorder (assessed using the Structured Clinical Interview [SCID-I]) and show a minimum symptom severity score of 14 on the HRSD or 21 on the BDI-II.

Every subject meeting the criteria is then seen by an experienced clinician, trained in psychoanalytic psychotherapy with adults, using the Tavistock Psychodynamic Interview (TPI). The research aspect of the instrument is described in detail below but it is also employed at this stage to assess the risk of suicide or of other psychiatric emergencies. As well, the interviewer seeks to identify any participant in whom the stress of psychotherapy might trigger a psychotic decompensation. Any such patient would be excluded on ethical grounds, although in the event no subject was excluded on this basis. No selection was made on the basis of any assessment of the subjects’ presumed suitability or unsuitability for psychoanalytic psychotherapy.

### Randomisation

The secure, automated telephone randomisation was provided by the Clinical Trials Unit at University College London. Minimisation is necessary to limit the impact of factors that might moderate treatment response. A gender bias has been identified in unipolar depression: UK prevalence is estimated at 28 per 1000 in women compared to 24 per 1000 in men [47]; pre-treatment severity has been shown to affect treatment outcome [48]; and antidepressant medications are the most frequent treatment for depression. Therefore, a computer-generated, adaptive minimisation algorithm incorporating a random element was employed to control for gender, depression severity (BDI-II of 21–39 or of 40+) and medication (on/off). Only the information needed for the minimisation algorithm was provided to the Clinical Trials Unit.

### The test intervention

As described, the PPD intervention is 60 fifty-minute sessions of once-weekly individual psychoanalytic psychotherapy. Each treatment therefore lasts for 18 months. The therapists are all senior clinicians of the Adult Department of the Tavistock Clinic. They have a qualification in one of the core mental health disciplines of psychology, social work, nursing or psychiatry, plus a British Psychoanalytic Council approved training in psychoanalytic psychotherapy or psychoanalysis. All therapy sessions, as well as the therapist peer-supervision sessions, are audio-recorded.

#### *The PPD treatment manual*

Taylor [49] has written a PPD treatment manual which describes the general principles of psychoanalytic psychotherapy, and the way in which they are applied to this patient group. The manual, which states the principles that should guide intervention, is permissive rather than prescriptive in its approach. Thus it authorises trained therapists to follow the direction of the patient’s narrative. In this the manual is representative of both psychoanalytic principles and current psychodynamic clinical practice [50]. The approach to manualisation of other evidence-based, longer term psychodynamic therapies employs a similar strategy [51].

As well as describing the aims and values of psychoanalytic treatment, the manual specifies the therapist’s tasks and purposes in relation to the TRD patient group. It outlines the kind of problems and conflicts found in this patient group; it emphasises the significance of the treatment framework, and it provides examples of session narratives (clinical material) typically encountered in depressed patients. The manual goes on to offer a number of psychoanalytic formulations of the expected components of depression, including the configurations underpinning chronicity and refractoriness. It illustrates the therapeutic approaches recommended in the opening, middle and ending phases of treatment. A specific section is devoted to management issues likely to arise with this patient group, including suicidal crises. The manual’s final section identifies those features which distinguish the PPD approach from other psychological interventions for MDD such as CBT. The manual has been adopted in a German multi-centre depression study [52].

#### *The assessment of treatment fidelity*

The PPD treatment is supported by fortnightly peer supervision and therapist workshops which are audio-recorded. The evaluation of treatment fidelity will use the Psychotherapy Process Q-Sort (PQS, [53,54]) a 100 item instrument distinguishing key actions, behaviours and therapists’ statements in different types of psychological therapy. Q-sort ratings are made according to a pre-determined normal distribution. This approach to the assessment of adherence has the advantage of being independent of the theoretical model of the treatment under evaluation. The degree of match of a given session to a psychoanalytic prototype can be quantified [55], and the absence of elements inimical to the test intervention confirmed. The instrument is valid. It has been widely used in similar investigations [56,57]. Trained raters independently score randomly selected samples of sessional material on a 1 to 9 scale in terms of resemblance to different psychological therapy prototypes. High levels of inter-rater reliability have been achieved [57-59] We expect the PPD treatment to score highly on the fostering of unstructured open-ended dialogue, use of transference interpretations, and the identification of unconscious processes. Low scores are expected for features of the CBT prototype such as the negotiation of foci on specified categories of thought and belief systems, encouragement of homework on particular issues outside the therapy and behavioural ‘experiments’.

### The treatment as usual condition

Patients randomly assigned to the TAU control group continue to receive treatment as directed by the referring practitioner. This may include referral for other specialist provisions. In the UK’s NHS, the range of such treatments is defined, and to an extent specified, in the treatment guidelines of the National Institute for Clinical Excellence [60]. Referral to psychoanalytic therapy is not within the guidance. Treatments and interventions received by the TAU control participants are recorded over the duration of the trial by using the Client Service Receipt Inventory (CSRI, [61]) and GP records. Arguments for and against this kind of comparison group are considered in the Discussion. At the end of their period of participation in the trial, the cases of all TAU participants are reviewed by the Tavistock’s Adult Department, with a commitment to offer PPD should it be indicated or requested.

### The outcome measures

#### *Primary outcome*

In psychotherapy and antidepressant medication outcome research, the Hamilton Rating Scale of Depression (HRSD, [44]) is the most widely used interview-based measure of depressive severity [62]. Independent, double-rated HRSD scores have been chosen as the trial’s primary index of depression severity. The HRSD is a structured interview which quantifies the severity of depressive symptoms in patients already diagnosed as suffering from depressive disorder. The psychometric properties of the instrument are acceptable [63]. In addition to categorical depressed/not depressed analyses (with a score of 14 as the cut-off point of remission), we intend to model the trajectory of scores across the 3.5-year observation period. This will permit the assessment of the severity of depression over time, with a particular focus on the 2-year follow-up period.

### Subsidiary outcomes

(a) **Self-report depression severity**: is assessed using the Beck Depression Inventory (BDI–II [64]) which is the most commonly used self-report instrument. It consists of 21 items, which yield a range of scores from 0 – 63. It has been shown to have excellent reliability (coefficient alpha of .92 for an outpatient population) and diagnostic efficiency [62,64].

(b) **Axis I disorders**: are assessed using the Structured Clinical Interview for DSM–IV, research version (SCID-I [65]). This semi-structured clinical interview assesses subjects on all five axes of DSM–IV diagnosis. The SCID-I, often considered the standard for clinical diagnoses [66], has acceptable inter-rater reliability (0.60 – 0.83; [67]). The diagnoses assessed in the TADS trial are MDD, dysthymia, alcohol abuse/dependency, drug abuse/dependency, anxiety disorders, OCD, and eating disorders.

(c) **Axis II personality disorders (PD)**: the Structured Clinical Interview for DSM–IV Personality Disorders Questionnaire (SCID-II-PQ [68]) is used to identify patients with probable Axis II disorders. This self-report measure has been found to have a 67% concordance with the SCID-II and strong associations have been reported between scores on the SCID-II and SCID-II-PQ for PD diagnostic concepts and categories (0.74 – 0.97 [only three under 0.80] [69]).

(d) **Personality functioning**: the information yielded by the Tavistock Psychodynamic Interview (TPI) allows Axis-II pathology to be assessed with the Shedler-Westen Q-Sort [42]. The information also permits the assessment of personality pathology according to the revised criteria of the proposed DSM-5. The TPI is described in more detail in the section below devoted to the qualitative part of the study.

(e) **Object relations**: the Person’s Relating to Others Questionnaire (PROQ2a [70]) is a 96-item self-report questionnaire which evaluates style of personal relating in terms of close (involving) vs. distant (seeking separation) and upper (relating from above downwards) vs. lower (relating from below upwards). The measure uses eight scales which are structured around these two axes. Birtchnell and Evans [71] have demonstrated that all scales have high internal validity (0.73 and above).

(f) **Social functioning**: the Global Assessment of Functioning scale (GAF, [72]) is a widely-used rating instrument which evaluates psychological, social and occupational functioning positioned on a hypothetical, 0 – 100 continuum of mental health - illness. It also comprises Axis V of the DSM-IV. GAF scores are made on the basis of the aggregated total of information available on the subject. A treatment trial similar to this one reported high inter-rater reliability (0.92) [72].

(g) **Subjective well-being**: is assessed using the Clinical Outcomes in Routine Evaluation – Outcome Measure (CORE-OM [73]). This 34-item self-report instrument assesses subjective well-being, symptoms, function, and risk. All domains show good internal reliability (of 0.75 – 0.95), convergent validity, and sensitivity to change [74]. The instrument has been widely used in the assessment of treatments offered by UK NHS Psychotherapy Departments [41].

(h) **Quality of life**: the Quality of Life Enjoyment and Satisfaction Questionnaire (Q-LES-Q [75]) is a self-report instrument consisting of 93 items grouped into eight quality of life areas - physical health, subjective feelings, work, household duties, school, leisure activities, social relationships, and general activities. Each item is rated on a 5-point scale of enjoyment/satisfaction over the previous week. Mean scores can be derived from the eight summary scales with a range from 0–100, with higher scores indicating better quality of life. The instrument achieves acceptable test–retest reliability (0.60 – 0.89). Its subscales show good levels of internal consistency (0.82 – 0.93 [76]).

(i) **Number of depression-free days**: at intake, and at all subsequent review/ follow-up interviews, participants are asked to estimate the number of depression-free days experienced in the preceding month

Table 2 lists all measures and the timing of their administration.

**Table 2:** Frequency of the TADS primary and secondary outcome measures

Time point	BDI	HRSD	CORE	PROQ2	QlesQ	SCID-I	SCID-II-PQ	CSRI
**Baseline**	✔	✔	✔	✔	✔	✔	✔	✔
**Reviews**								
3 Month	✔	✔	✔	✔	✔			
6 Month	✔	✔	✔	✔	✔			✔
9 Month	✔	✔	✔	✔	✔			
12 Month	✔	✔	✔	✔	✔			✔
15 Month	✔	✔	✔	✔	✔			
18 Month	✔	✔	✔	✔	✔	✔	✔	✔
**Follow-up:**								
6mFU	✔	✔	✔	✔	✔	✔	✔	✔
12mFU	✔	✔	✔	✔	✔	✔	✔	✔
24mFU	✔	✔	✔	✔	✔	✔	✔	✔

### The analysis of data

To preserve neutrality, no data will be analysed until all subjects have reached the six month follow-up point. An intention-to-treat analysis will be performed in the first instance (i.e. analysing all available data from randomised participants) using for all analyses the STATA, version 11.0 package. The numbers and percentages of losses to follow-up at 12, 18, and 24 months after randomisation will be reported and will be compared between the treatment arms with absolute risk differences (95% CIs). Any deaths and their causes will be separately reported. To increase the power and precision of the estimated treatment effect, missing baseline covariates will be imputed for our main analysis using either mean or regression imputation [77]. All available results will be used without missing outcomes being imputed. Primary analyses will be based on the assumption of ignorable drop-outs. In secondary analyses, missing values will be replaced by multiple imputations using Markoff Chain Monte Carlo methods [78].

For continuous outcome variables, the differences in mean score between those randomised to either arm of the trial will be examined using analysis of covariance adjusting by 1) baseline value of outcome 2) baseline value of the outcome, gender and age. The assumption of linearity will be assessed by residual analysis; if necessary, bootstrapping techniques will be employed. Sensitivity analyses will be conducted to take into account missing data using multiple imputation, which assumes data are 'missing at random'. For continuous response variables, statistical analyses will be based on linear mixed models [79].

For binary responses, mixed-model logistic regression models and generalized estimating equations will be used [80]. Logistic regression will be used to compare proportions of patients that have lost their diagnosis during the treatment and follow-up period in the two groups using an HRSD of 14 as criterion. Analysis of 'time to first recovery' and ‘time to first relapse’ outcome will also be conducted using survival analysis methods and the log-rank test for bivariate comparisons and Cox's proportional hazards to adjust for gender, stratification variables and time spent on treatment prior to randomisation. The dependencies between design points in the case of linear mixed models will be accounted for by assuming an unstructured correlation structure.

Statistical significance will be tested with the Wald test, and CIs will be established by the Delta method [81]. Time will be handled as a categorical variable with five possible values - 0, 6, 12, 18, 24 months. The basic model will include the main effects of time, of treatment group, of the difference between theoretical and realised date of measurement and first order interaction of time and treatment. A complete model will include potential confounding factors (e.g. age group, gender etc). Complementary analyses will be carried out adjusting for the baseline level of outcome measures. 'As treated' models will be carried out by including variables describing compliance (e.g. waiting time from randomisation to initiation, degree of participation, including an indicator whether the patient received the study treatment) and auxiliary treatments such as medication.

### Economic evaluation

The exact number of therapy sessions is recorded. Costs of these will be estimated using data on staff salaries, overheads and activity levels. Use of other services during the 12-month period before baseline and during the follow-up (broken down into six-month periods) is measured with the Client Service Receipt Inventory (CSRI, [61]) which asks for information on the number and duration of contacts with primary and secondary health and social care professionals, and time lost from work. Service costs are calculated by combining the service use data with standard unit costs [61,82]. Lost employment costs will be calculated by combining time off work with average wage rates. Access to medical notes is agreed. These will be used to supplement CSRI data. Given that under-reporting of service use is more likely than over-reporting, we will use the higher number of contacts with clinicians if the two sources differ. Cost-effectiveness will be assessed by combining cost data with the HRSD change score: incremental cost-effectiveness ratios will be computed to show the extra cost incurred to achieve a one-unit improvement on the HRSD; uncertainty around these estimates will be addressed using cost-effectiveness planes. These analyses will be conducted first for healthcare costs and then for total costs (i.e. use of other services and lost employment). Sensitivity analyses will be conducted by increasing/decreasing the therapy costs by 25% and by using minimum wage rates to value lost employment.

## Qualitative & psychoanalytic case-study evaluation

### The private theories of participant and therapist

The Private Theories Interview (PTI, [83]) has been used in the investigation of patients’ views of illness and treatment response [84-86]. The imposition of the researcher’s constructions is minimised to permit the interviewee’s views to emerge. Here, we use this semi-structured interview to collect narrative material relevant to participants’ theories about their depression, their experience of treatment and of participation in a long-term, randomised trial such as this. This is done with a purposive sub-sample drawn from both patient groups and in the case of the PPD arm, with the corresponding therapists. The sample will also be matched for age, sex, demographics and treatment-response (completers versus drop-outs; and responders, intermediate or non-responders). Analysis is systematic and follows a coding manual [83] organised around the phenomenological principles of “categorization of meaning” and “concentration on meaning” [87]. Subsequent analyses will be concerned with the subject’s perspective on any therapeutic change and any associations between participants’ views and the quantitative outcome measures.

### The Tavistock psychodynamic interview (TPI)

The TPI is a specially designed instrument [88] administered by a trained psychoanalytic clinician. It draws on well validated psychodynamic and attachment-based interviews. These are the Adult Attachment Interview (AAI; [2]), the Current Relationships Interview (CRI; [89]), and the Quality of Object Relating Scale (QORS; [90]). The AAI [91], is based upon the way that the formal quality of adult discourse has been found to be lawfully related to attachment patterns established in early development. There is strong evidence for thematic continuity between narratives, dreams, early autobiographical memories and attachment patterns [92]. The CRI and the QORS both aim to assess the quality of object relationships and have been shown to have good validity [93].

The interview is carried out at the subject’s entry to the trial (TDI-Initial) and repeated two years later (i.e. six months after the end of therapy in the case of patients in the PPD condition). In addition to the clinical and personality assessment functions already described, the TPI collects narrative data about subjects' relationship histories. Particular attention is paid to key developmental and transitional phases in early childhood, adolescence, adulthood. Subjects are asked to relate a recent dream and their earliest memory. The aim is to capture subjects’ representations of key interpersonal relationships along with psychodynamically important aspects of cognitive and emotional processing, to arrive at an independent psychodynamic formulation of the subject’s illness.

The end version of the TDI looks for changes in the psychological and interpersonal functions which according to psychoanalytic thinking play a part in depressive disorders. Any changes in personal narratives or in functioning across the domains of early and later relationships, work, physical health, etc. are noted. The initial interview’s request for a recent dream and an earliest childhood memory is repeated verbatim to compare participants’ responses at the different time points. Independent judges will then categorise subjects as responders, intermediate or non-responders in terms of these psychodynamic variables. This categorisation will then be correlated with treatment allocation and quantitative outcome data. Case vignettes will delineate prototypes to examine the possibility of different constellations of illness, types of vulnerability and different patterns of responsiveness/non-responsiveness.

### Psychoanalytic case-study methods

Here we detail three sub-studies based on psychoanalytic theory, and which use case-study methods. They are designed to provide a general model which may help explain the RCT findings, and to probe specific questions.

#### A psychoanalytic typology of different forms of chronic depression

Applying the ideas of Rosenfeld [94], Blatt [95], Blatt & Zuroff [96] and Bleichmar [97], along with the preliminary clinical investigation of five pilot cases, a provisional typology consisting of seven components/dimensions has been constructed [98,99]. A purposive sub-sample drawn from the patients in the PPD arm (N = 67) will be selected by members of the clinical research workshops to include the full range of the most frequently encountered sub-forms of TRD, plus any exceptions, extreme types or others thought to be especially informative [100,101]. It will contain a minimum of 25 cases. On the basis of extended clinical discussions of therapists’ verbal session reports over the course of the subjects’ treatments, plus transcripts of audio-recordings, an evaluating group of therapists will assess the model’s fit, and its ability to make sense of the heterogeneity in the sample. Each subject will then be described along with a proforma ‘rating’ based upon the typology. The research group’s consensus and description will be checked against the therapist’s judgement, and if necessary will be modified by it (see [102,103]). The original accounts of the sub-set and the typology descriptions will be compared and checked against the therapists’ experience of the whole PPD sample. Any modifications of the model which seem to be required will then be set out. The aim throughout will be to provide ‘thick descriptions’, each of which will be referenced to the different kinds of information used.

#### The nature of change or non-change in PPD treated patients

Using a similar approach a subset of the PPD arm will be selected to include a full range of outcome categories - responders, non-responders, intermediate and treatment failures - determined on the basis of therapists’ clinical judgements. Therapists will then provide ‘thick descriptions’, again referenced to the information used, to detail the elements of the positive or negative change occurring. These descriptions will be employed with the quantitative findings and the TPI to triangulate a more three-dimensional picture of different outcomes.

#### Investigating the possible role of an increased sense of personal agency in PPD responders, and of persisting passivity in non-responders

There is evidence that a sense of personal agency [104], as one part of the executive function of the personality [105] plays an important part in mental health [106,107], in developmental or social resilience [108], and in recovery from mental disorder. In the case of depression, variations in psychosocial functionality correlate poorly with symptom severity and variations in motivation cannot be accounted for on the basis of mood alone. Psychoanalytic findings suggest that persistent forms of passivity, which may also be encountered in subjects with TRD, may militate against treatment response [109,110]. In this part of the qualitative study, the therapist selected sample of responders, non-responders, intermediate and treatment failures described above will be evaluated in terms of a psychoanalytic conceptualisation of personal agency devised by Amino & Taylor (2008, unpublished).

### Ethical approval

The trial received ethical approval from the NHS West Midlands Research Ethics Committee on 28 May 2002 – reference number MREC02/07/035. Amendments were approved in July 2005, October 2009, and February 2010. Some of the ethical issues encountered in the conduct of this long-term trial are considered in the Discussion below.

Trial progress and participant recruitment. See: (Additional file 1: Figure S1).

## Discussion

This pragmatic RCT is notable in that through a high-quality design it evaluates a previously under-researched treatment, namely medium to long-term psychoanalytic psychotherapy, which aims to reduce the underlying vulnerability to depression in people suffering from a particularly complex and enduring form of this disorder. Currently, there is only one trial with a group of patients of a roughly comparable complexity [111-114]. A positive finding in our trial would significantly improve the options available for patients with this expensive and relatively poorly-served condition, which also represents a considerable burden worldwide [60,115].

However, as we have argued, the features of both the disorder and the treatment strain the limits of methodology in classical randomised clinical trials. We decided that the trial should be ecological, namely that it ought to offer information about the actual outcome over the medium to long-term of a clinically significant patient sample in the NHS. Many of the trial’s design choices followed from this decision. In this section, we discuss how we have tried to mitigate some of the strains, and list some inevitable limitations.

### The patient sample

In relation to the diagnosis of depressive and other common mental disorders, we do not yet know how to carve nature at the joints. If our study were to meet its primary requirement of ecological validity, the sample selected had to involve the fewest possible assumptions. We therefore defined the sample operationally, and included participants with co-morbid Axis I and II disorders as well as problems with physical health. Inevitably, this leads to a heterogeneous sample that includes many unknown variations capable of affecting both outcome and the interpretation of findings. Although PPD has the advantage as a treatment of being adaptable to each individual’s psychopathology, we still expect a large amount of residual variation in responsiveness amongst different patients. The treatment under test will help some participants more than others, and these variations may cancel each other out [116]. In this context, examining mean outcomes, for example, will be insufficient, and while modelling trajectories of outcomes using mixed-effects growth curves allows for differential treatment responses to be investigated, this is only possible when the critical moderators involved first have been identified and then measured. In the absence of predictions derived from well-founded theories of pathology, this is a hit or miss way of proceeding. The poor performance of outcome research in answering the question “what works for whom?” suggests that this approach might be misconceived [117]. It may be necessary to be able to drill down through group data to discover what may be deeper structures in the individual in order to understand the basis of differential degrees of responsiveness/non-responsiveness. Thus we have collected qualitative data using both psychoanalytic and phenomenological methods.

### The treatment as usual condition

A TAU control condition was chosen because it is an ecologically sound comparison group. TAU control conditions have been widely used in outcome trials, although their rationale, and what any given TAU condition may involve, has varied from case to case [25]. Our TAU control condition is only partly determined by the management protocols for these patients specified in NICE guidelines. In actual practice, there are widespread variations in the mental health resources available in different locations (NHS Atlas) and, in our experience, these are especially marked in the treatment that TRD patients receive. Some may receive exemplary care through their GPs, including social support, community mental health teams, brief counselling, and pharmacological and psychological interventions of appropriate types, durations and intensities. Others may get little if any help beyond token pharmacological treatment, with little case management. Some, perhaps following many failed attempts at treatment, may have withdrawn from all treatment endeavours. They feel they have exhausted all treatment possibilities and end up quite seriously neglected and deprived. As we have also suggested, certain characteristic features of psychosocial functioning may be integral to the TRD disorder. By adversely affecting important help-seeking behaviours, these actively exert negative effects on service provisions. These considerations lend weight to the decision to study this naturally occurring control and comparison condition [118].

A long-term trial involving patients with severe depressive disorders operates against a background of a continuous level of chronic suicidal risk punctuated by exacerbations and crises. Our guiding principle is that the TAU participant’s clinical needs must always come first. The senior research clinician and the trial’s clinical director explicitly assume responsibility for participants’ safety and have the authority to intervene and mediate in a participant’s care. In most instances this does not conflict with one of the needs of research - for instance, to keep as many subjects in the trial as possible for as long as possible. However, it follows that the trial’s TAU condition is not always and not only Treatment As Usual. Moreover, the consistent, reliable contact involved in the researcher’s administration of research measures necessarily functions like a low-intensity, psychodynamically informed supportive intervention.

### Randomisation

TRD participants vary in their resilience to the significant stresses that research involves. One of the most potent stresses is randomisation powerfully stirs up feelings of rejection especially in those who already have histories of severe loss or abandonment. Subjects who had strong wishes for the PPD treatment feel disappointed, hurt or angry when the allocation is not that for which they hoped. These subjects may then drop-out. Others drop out because of their fears about what treatment may involve. When participants are told of their treatment allocation, it is important that the research staff show sensitivity to the situation. The psychoanalytically-trained research clinicians allow space in the initial clinical interview for the subject to express how they felt about the allocation they received. As well, the research clinician has to be mindful of the underlying feelings aroused by the long research process especially with those who were randomised to the TAU control.

### The need for best research practice

Over the longer-than-usual time of this trial, we considered there might be a greater tendency for subjects to drop out. To combat this, continuity and absolute best practice in the research team need to be ensured. Researchers needed to be reliable, persistent, flexible, sympathetic and responsive with all the subjects. Newsletters and other communications about the trial can be used to help subjects remain aware of the fact that they are helping in a valuable endeavour.

However, most research staff are not clinically qualified and are often at the beginnings of their career. It is therefore essential that these research staff are provided with sufficient clinical supervision by qualified research clinicians. The supervision has three functions. First, it gives the research staff a chance to process the considerable emotional impact associated with the way that subjects often powerfully convey their feelings. Second, it helps researchers to reconcile subjects’ ongoing communication of their needs with the researcher’s task of equipoise, while still helping subjects stay in the trial. Third, it gives the research clinician an opportunity to keep in touch with each patient’s condition.

### The limitations of the trial

When compared with other long-term psychoanalytic psychotherapy (LTPP) outcome studies our N of 129 may be regarded as large, but considered statistically the trial is somewhat under-powered. Unfortunately, the very large and expensive treatment service resources necessary for a significantly bigger sample are simply not available. A second limitation is to be found in the impossibility of concealing treatment allocation from research interviewers. We seek to mitigate this by double-rating the primary outcome measure using independent raters working with anonymised recordings. Another limitation is the inherent variability both of our test treatment and of the TAU comparison condition. Although our design includes a treatment manual, assessment of adherence, and careful recording of the TAU care control patients actually receive this still does not permit the kind of quasi-experimental assessment of the impact of the components that are possible with less complex treatment procedures. Finally, in a long-term trial with patients of this kind the inevitably high rate of attrition could easily cause a statistically unacceptable standard mean error [119].

In this discussion we have considered only some of the issues in long-term randomised trials of complex treatments for complex mental health conditions. We opted for this composite, ecological design as a first stage study in which a standard RCT was complemented by evaluative methods in order to produce in-depth models helpful in understanding the condition itself, as well as with the development of more optimal treatment and case management. We want to emphasise the importance of research procedures capable of meeting the challenges posed by the disorder. Some knowledge of the complex nature of the disorder is also necessary to properly evaluate both the structure of the research, and its eventual findings.

## Competing interests

DT & PF are training and supervising psychoanalysts of the British Psychoanalytic Society.

## Authors’ contributions

PF, FR, RT & DT each contributed to writing this paper. PR, DT, & JC were responsible for the concept and design of the trial; DT wrote the PPD treatment manual; PR & JC were responsible for choice of measures; JC designed the TPI and initial PPD adherence study; PR & SM were responsible for trial ethical approval, and SM with JC for initial recruitment and for liaison, training, and SM for a mid-trial data audit; JC & SM have been responsible for day-to-day administration of the trial, a post now held by FR who is responsible for second phase recruitment, amendment to research ethics, and design & delivery of Private Theories qualitative study; RT administers TPI’s & their data, provides clinical consultations to all Trial patients, supervision of researchers & liaison with clinicians; PF & DT are jointly responsible for the delivery of the Trial. PF is the TADS Principal Investigator and has overall responsibility for research management. All authors read and approved the final manuscript.

## Pre-publication history

The pre-publication history for this paper can be accessed here:

http://www.biomedcentral.com/1471-244X/12/60/prepub

## Supplementary Material

Additional file 1 **Figure S1.** TADS Consort Diagram: recruitment and treatment allocation. Click here for file
