# Supplementary figures and images for: Tavistock Adult Depression Study (TADS): a randomised controlled trial of psychoanalytic psychotherapy for treatment-resistant/treatment-refractory forms of depression
Source: BMC Psychiatry. 2012 Jul 12;12:60. doi: 10.1186/1471-244X-12-60 (PMC3395560; doi:10.1186/1471-244X-12-60)

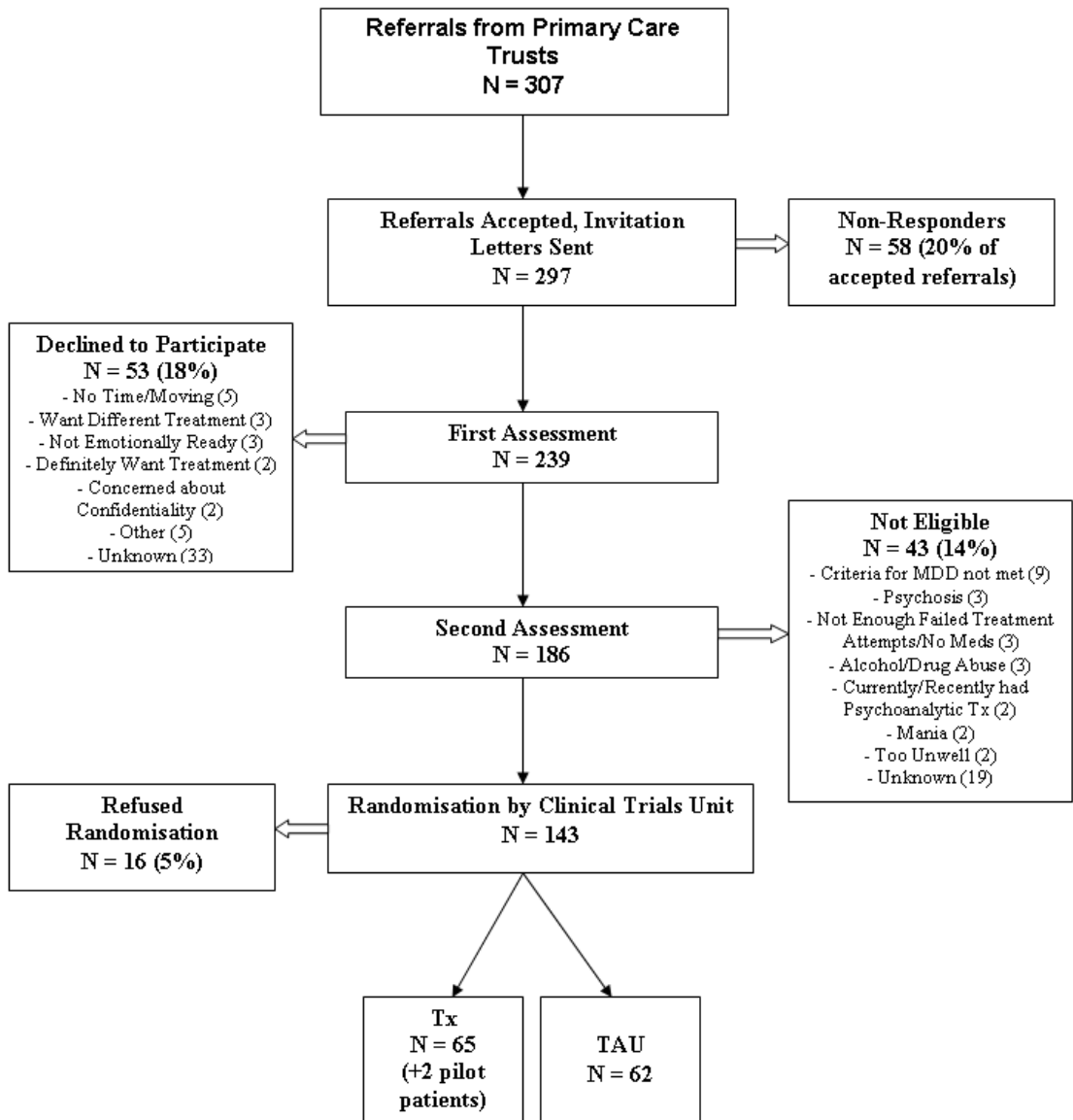

Figure 1

Supplement: Additional file 1 — Figure S1. TADS Consort Diagram: recruitment and treatment allocation. [file 1471-244X-12-60-S1.pdf]
